# Comparison of analytical and clinical performance of CLART HPV2 genotyping assay to Linear Array and Hybrid Capture 2: a split-sample study

**DOI:** 10.1186/s12885-015-1223-z

**Published:** 2015-04-02

**Authors:** Ditte Møller Ejegod, Matejka Rebolj, Jesper Bonde

**Affiliations:** 1 Department of Pathology, Copenhagen University Hospital, Allé 30, 2650, Hvidovre, Denmark; 2 Department of Public Health, University of Copenhagen, Copenhagen, Denmark; 3 Clinical Research Center, Copenhagen University Hospital, Hvidovre, Denmark

**Keywords:** Cervical cancer, Human papillomavirus, Genotyping, Linear array, CLART, Hybrid capture 2

## Abstract

**Background:**

Human Papillomavirus (HPV) genotyping has an increasingly important role in cervical cancer screening and vaccination monitoring, however, without an internationally agreed standard reference assay. The test results from the most widely used genotyping assays are read manually and hence prone to inter-observer variability. The reading of test results on the CLART HPV2 genotyping assay is, on the other hand, automated. The aim of our study was to directly compare the detection of HPV genotypes and high-grade cervical intraepithelial neoplasia (CIN) by CLART, Linear Array (LA), and Hybrid Capture 2 (HC2) using samples stored in SurePath.

**Methods:**

Residual material from 401 routine samples from women with abnormal cytology was tested by CLART, LA, and HC2 (ClinicalTrial.gov: NCT01671462, Ethical Committee approval: H-2012-070). Histological outcomes were ascertained by linkage to the Danish nation-wide Pathology Data Bank. For comparison of CLART and LA in terms of genotype detection, we calculated κ-coefficients, and proportions of overall and positive agreement. For comparison of CIN detection between CLART, LA, and HC2, we calculated the relative sensitivity and specificity for high-grade CIN.

**Results:**

The κ-coefficient for agreement in detection of genotypes 16, 18, 31, 33, 35, and 51 was ≥0.90 (overall agreement: 98-99%, positive agreement: 84-95%). The values were slightly lower, but still in the “substantial” range for genotypes 39, 45, 52, 56, 58, 59, and several low-risk genotypes. The relative sensitivity of CLART for ≥ CIN2 and ≥ CIN3 was not significantly lower than that of LA and HC2, although CLART showed a higher specificity than HC2.

**Conclusions:**

In Danish women with abnormal SurePath cytology, CLART and LA were highly comparable for detection of most high-risk and low-risk genotypes; and CLART’s sensitivity for high-grade CIN was comparable to that of both LA and HC2.

## Background

Cervical cancer is caused by high-risk Human Papillomavirus (HPV) genotypes, whereas low-risk genotypes cause benign lesions [1-3]. Genotyping of HPV infections has an increasing role in cervical screening and vaccination monitoring [4,5], however, without an internationally agreed standard reference HPV genotyping assay [4]. With more than 100 genotyping assays on the market, the question remains: which genotyping assays have the requisite validation data to support their use. The two most widely used, Linear Array (LA; Roche Diagnostics, Pleasanton, CA), and INNO-LiPA (Fujirebio Europe, Ghent, Belgium), detect 37 and 28 genotypes, respectively, and are typically read manually and hence prone to inter-observer variability in reporting test results. Papillocheck (Greiner Bio-One, Frickenhausen, Gemany), on the other hand, detects 24 genotypes, and uses automated reading [6-8]. In contrast to these commercially available genotyping assays, the GP5+/6+ polymerase chain reaction (PCR) followed by enzyme immunoassay is an in-house assay and its performance may be laboratory-dependent.

CLART HPV2 (CLART; Genomica, Madrid, Spain) is a commercially available PCR-based genotyping HPV DNA assay, based on genotype amplicon-specific hybridization on a microarray. The assay has two internal controls, a DNA control (human CTFR gene) for sample sufficiency, and an amplification control (plasmid) for process control in each tube. It detects 35 genotypes: the 13 high-risk (16, 18, 31, 33, 35, 39, 45, 51, 52, 56, 58, 59, 68) [1] and 22 low-risk (6, 11, 26, 40, 42, 43, 44, 53, 54, 61, 62, 66, 70, 71, 72, 73, 81, 82, 83, 84, 85, 89). Detection of individual genotypes was calibrated against known copies of cloned plasmids. Essential for high-throughput screening settings, the reading of test results is automated. Furthermore, CLART can be applied to several sample types, including formalin-fixed paraffin-embedded specimens [9,10]. Several laboratories participated with CLART in the WHO HPV LabNet Proficiency Studies [4,11], emphasizing that while it is rarely described in scientific publications [12-15], it is frequently used in clinical, non-research laboratories.

Here, we compared the analytical and clinical characteristics of CLART to those of LA and Hybrid Capture 2 (HC2; Qiagen, Gaithersburg, MD) in Danish women with abnormal cytology.

## Methods

The data presented in this study were partially collected within the Danish arm of a European CE-IVD trial evaluating a new molecular HPV assay (ClinicalTrials.Gov ID: NCT01671462). From this trial, test results on HC2 and LA were used here, whereas the CLART HPV2 testing was undertaken specifically for the purpose of the current study. Residual material from 411 consecutive, unselected SurePath samples with abnormal cytology (atypical squamous cells of undetermined significance or worse, ≥ASCUS) were collected from up to 10 routine racks per day processed in the laboratory between September and October 2012. After the samples had been collected, we excluded those with insufficient quantity, ≤1.0 ml, of the residual SurePath material available post the routine cytology. Of the collected samples, 10 were excluded due to this criterion.

Cytology evaluation was undertaken by Focal Point assisted screening (BD, Burlington, NC). Slides were read by cytoscreeners, with abnormal findings adjudicated by pathologists and reported using the Bethesda 2001 system. Women aged ≥30 years with ASCUS had routine reflex HC2 HPV triage. After a negative HC2 test result, any initial ASCUS diagnoses were routinely downgraded to normal cytology, with women being referred back to routine screening. Other cytology reading was undertaken blinded to HPV testing. Women with HC2-positive ASCUS were referred for colposcopy, as were women with high-grade squamous intraepithelial lesions (HSIL), atypical squamous cells – cannot exclude HSIL (ASC-H), atypical glandular cells (AGC), adenocarcinoma in situ (AIS), cytological squamous carcinoma, and women with persistent ASCUS at age <30 years or low-grade squamous intraepithelial lesions (LSIL). Women with ASCUS at age <30 years or LSIL had repeated cytological testing. Follow-up tests until end of February 2014 were retrieved from the Danish National Pathology Data Bank (Patobank; [16]). Cases of cervical cancer were adjudicated, based on the free text in the Patobank, by an expert pathologist from the same laboratory.

### HPV testing

One-half ml of SurePath sample material was centrifuged for five minutes at 14,000 revolutions per minute. Cell pellets were re-suspended in a mix of 180 μl phosphate buffered saline (10× conc. pH 7.4, Pharmacy product) and 20 μl Proteinase K (Roche Diagnostics, Rotkreuz, Switzerland). Samples were vortexed and incubated for one hour at 56°C and one hour at 90°C. DNA was purified using MagNa Pure LC 96 instrument with MagNa Pure LC Total Nucleic Acid Isolation Kit (Roche Diagnostics). Aliquots of extracted DNA were used for both CLART and LA testing. On average, samples were DNA extracted 17 days (range 10–27) after having been received in the laboratory. Extracted DNA was stored frozen until LA and CLART testing.

PCR amplification was performed using CLART HPV2 Amplification kit (Genomica). Five μl of purified DNA were used as template per reaction. Prior to visualization, the PCR products were denatured at 95°C for 10 minutes. Hybridization was performed using 10 μl of the denatured PCR products on the CLART microarray, and subsequent visualization was done according to manufacturer’s specifications. The genotyping results were analyzed and reported automatically on the Clinical Array Reader (Genomica).

LA detects the 13 high-risk, and 24 low-risk genotypes. The assay has an internal human β-globin control for sample sufficiency and assay performance. With the final volume of 50 μl, 12.5 μl of purified sample DNA and 4 μl of purified control DNA were added for each sample and control PCR reaction. PCR was performed on GeneAmp PCR system 9700 (Applied Biosystems, Foster City, CA). Twenty-five μl of PCR reaction were used for LA testing according to the manufacturer’s protocol. The results on the LA strips were read independently by DME and a scientific assistant. In case of disagreement, consensus was sought. The difference in the DNA input per test between LA and CLART reflects the manufacturer specifications.

HC2 analysis was undertaken on the SurePath post-quot material, in concordance with the manufacturer’s specifications. Samples were denatured manually prior to the analysis on the manual HC2 Modular system (Qiagen, Gaithersburg, MD, USA). On average, samples were denatured 17 (range: 3–23) days after having been received in the laboratory and stored according to the manufacturer’s recommendations prior to HC2 testing.

### Statistical analyses

A sample was considered high-risk positive for HPV if at least one high-risk genotype was detected, and low-risk positive when at least one of the remaining 18 genotypes detectable by both assays (6, 11, 26, 40, 42, 53, 54, 61, 62, 66, 70, 71, 72, 73, 81, 82, 83, 84) was detected without any high-risk genotype. CLART automatically reports genotypes separately when they are detected in an “uncertainty” range, i.e. with weak signals. Reflecting routine practice in our facility, these genotypes are considered positive only if part of multiple infections. The same definition was used for LA in case of bands with weak signal intensity. A positive HC2 test result was defined as RLU/CO value ≥1.0.

Differences in the distribution of women’s characteristics for the three assays were calculated using the *Χ*^2^ distribution. For all 31 HPV genotypes detectable by both assays, we calculated the κ-coefficients and proportions of overall and positive agreement. κ-coefficients >0.60 were considered to indicate “substantial” agreement [17]. Overall agreement was calculated as the proportion of all samples that returned the same test result on both CLART and LA (no genotype, or same genotype). Positive agreement was calculated as conditional probability that both assays detected a particular genotype if at least one did. The proportion of high-grade CIN (≥CIN2 or ≥ CIN3) with a positive test result on a particular HPV assay was used as an indicator of the assay’s clinical sensitivity. As an indicator of clinical specificity, we calculated the proportion of women testing negative among those without high-grade CIN; we assumed that women with cytology but without histology in follow-up had no high-grade CIN, and excluded women who were lost to follow-up. The 95% confidence intervals (CI) for sensitivity and specificity were calculated using binomial distribution. We calculated the relative clinical sensitivity and specificity for CLART by comparing its sensitivity and specificity to LA and HC2. The 95% CI for relative sensitivity and specificity, and for the relative prevalence (RP) of genotypes (CLART vs. LA), were calculated assuming that their logarithms were approximately normally distributed.

### Ethical approval

LA and HC2 data were collected with informed consent as part of the Danish arm of a multicenter European trial (ClinicalTrial.gov Identifier: NCT01671462), approved by the Danish Capital Region Ethical Committee (H-2012-070). Informed consent was obtained by the sample taking gynecologists, and maintained in the women’s patient records, as well as in copy at the Department of Pathology in concordance with Danish Ethical guidelines. Additional testing on CLART, not used for clinical management, was undertaken as a quality development study, for which ethical approval and informed consent are not required, in concordance with the current Danish law.

## Results

The 401 women were aged 17–78 years (mean 32.8, median 29). Most (N = 357, 89%) were in the screening age (23–65 years). ASCUS was diagnosed in 103 (26%) women, 161 had LSIL (40%), 30 ASC-H (7%), 106 HSIL (26%) and one (<1%) had cytological signs of carcinoma (Table 1). On average, women were followed for 17 months (range: 506–542 days). Seventeen (4%) were lost to follow-up. On CLART and LA, the proportion of high-risk genotypes decreased by age and increased by the severity of the cytologic interpretation; on HC2, the trends were not statistically significant. The differences between CLART and LA were not statistically significant. Between CLART and HC2, some differences were seen, particularly by age where more women aged ≥30 years had high-risk HPV genotypes detected on HC2 than on CLART. The differences in the distribution of test results in women lost to follow-up were not statistically significant.

**Table 1:** **Description of the 401 women included in the study**

	Total	CLART			LA			HC2		P	
		High-risk genotypes	Low-risk genotypes	No HPV genotypes^c^	High-risk genotypes	Low-risk genotypes	No HPV genotypes^c^	Positive test result	Negative test result	CLART vs. LA	CLART vs. HC2
**Total**	401 (100%)	311 (78%)	54 (13%)	36 (9%)	326 (81%)	54 (13%)	21 (5%)	355 (89%)	46 (11%)		
**Age (years)**											
<30	215 (100%)	180 (84%)	21 (10%)	14 (7%)	184 (86%)	21 (10%)	10 (5%)	192 (89%)	23 (11%)	0.701	0.090
30-39	96 (100%)	74 (77%)	16 (17%)	6 (6%)	80 (83%)	13 (14%)	3 (3%)	86 (90%)	10 (10%)	0.462	0.020
≥40	90 (100%)	57 (63%)	17 (19%)	16 (18%)	62 (69%)	20 (22%)	8 (9%)	77 (86%)	13 (14%)	0.210	0.001
P		0.001			0.012			0.602			
**Cytology**											
ASCUS	103 (100%)	78 (76%)	11 (11%)	14 (14%)	82 (80%)	10 (10%)	11 (11%)	88 (85%)	15 (15%)	0.776	0.078
LSIL	161 (100%)	113 (70%)	37 (23%)	11 (7%)	119 (74%)	38 (24%)	4 (2%)	140 (87%)	21 (13%)	0.180	<0.001
≥HSIL^a^	137 (100%)	120 (88%)	6 (4%)	11 (8%)	125 (91%)	6 (4%)	6 (4%)	127 (93%)	10 (7%)	0.455	0.156
P		<0.001			<0.001			0.156			
**Worst follow-up outcome**											
No follow-up	17 (100%)	14 (82%)	2 (12%)	1 (6%)	14 (82%)	3 (18%)	0 (0%)	16 (94%)	1 (6%)	0.549	0.287
Normal cytology or negative HPV test	75 (100%)	56 (75%)	8 (11%)	11 (15%)	60 (80%)	6 (8%)	9 (12%)	60 (80%)	15 (20%)	0.732	0.435
Abnormal cytology or positive HPV test	10 (100%)	7 (70%)	2 (20%)	1 (10%)	8 (80%)	2 (20%)	0 (0%)	8 (80%)	2 (20%)	0.587	0.605
Inadequate histology	9 (100%)	3 (33%)	3 (33%)	3 (33%)	3 (33%)	3 (33%)	3 (33%)	6 (67%)	3 (33%)	1.000	0.157
CIN0	98 (100%)	57 (58%)	29 (30%)	12 (12%)	61 (62%)	31 (32%)	6 (6%)	77 (79%)	21 (21%)	0.333	0.002
CIN1^b^	67 (100%)	58 (87%)	5 (7%)	4 (6%)	60 (90%)	5 (7%)	2 (3%)	65 (97%)	2 (3%)	0.704	0.028
CIN2	35 (100%)	32 (91%)	2 (6%)	1 (3%)	33 (94%)	2 (6%)	0 (0%)	34 (97%)	1 (3%)	0.602	0.303
CIN3	86 (100%)	81 (94%)	3 (3%)	2 (2%)	83 (97%)	2 (2%)	1 (1%)	85 (99%)	1 (1%)	0.757	0.096
Cervical cancer	4 (100%)	3 (75%)	0 (0%)	1 (25%)	4 (100%)	0 (0%)	0 (0%)	4 (100%)	0 (0%)	0.285	0.285
P		<0.001			<0.001			<0.001			

*Abbreviations:ASCUS* atypical squamous cells of undetermined significance, *CIN* cervical intraepithelial neoplasia, *HPV* human papillomavirus, *HSIL* high-grade squamous intraepithelial lesions, *LSIL* low-grade squamous intraepithelial lesions.

^a^Including atypical squamous cells – cannot exclude HSIL, adenocarcinoma in situ, atypical glandular cells, cytological signs of carcinoma.

^b^Including histological atypia and CIN not otherwise specified.

^c^Or HPV genotypes not detectable by both CLART and LA (CLART: 43, 44, 85, 89; LA: 55, 64, 67, 69, IS39, CP6108).

### Detection of HPV genotypes

In total, 311 (78%) women had high-risk genotypes on CLART, and 326 on LA (81%; RP: 0.95, 95% CI: 0.89-1.02). CLART detected statistically significantly fewer HPV 39, 45, 54, 62, and 73 infections than LA, whereas LA detected fewer HPV 58 and 82 (Table 2). For HPV 16, 18, 31, 33, 35, and 51, the agreement between CLART and LA was excellent (κ ≥ 0.90, overall agreement 98-99%, positive agreement 84-95%). For HPV 39, 45, 52, 56, 58, and 59, the agreement was substantial (κ ≥ 0.60, overall agreement 94-96%, positive agreement 46-64%); however, for HPV 68, the agreement was poor (κ = 0.26, overall agreement 93%, positive agreement 17%). For the 18 low-risk HPV genotypes detectable by both genotyping assays the agreement was in general good, although for genotypes HPV 54, 62, 73, and 82, the agreement was poor (κ < 0.60, overall agreement 93-96%, positive agreement 35-42%). However, these genotypes and HPV 68 were not highly prevalent in this population. This was similar in 125 women with ≥ CIN2 (treatment threshold in Denmark), with CLART detecting statistically significantly fewer HPV 45 infections than LA, RP: 0.35 (95% CI: 0.14-0.87; Table 3). CLART found single HPV infections in 130 (32%), and multiple infections in 235 (59%) women (Table 4). For LA, this was the case in 121 (30%) and 259 (65%), respectively. These differences were not statistically significant, RP for single infections: 1.07 (95% CI: 0.87-1.32). The κ was 0.64, with an overall agreement of 81% (95% CI: 77–85). For detecting high- and low-risk infections (Table 5), the κ was 0.76, with an overall agreement of 92% (95% CI: 88–94), and positive agreement (for detecting at least one high-risk genotype) of 92% (95% CI: 89–95). The differences in detecting high-risk infections overall (for detecting at least one high-risk genotype) were not significant, RP: 0.95 (95% CI: 0.89-1.02).

**Table 2:** **Detection of individual high-risk and low-risk HPV genotypes by CLART and LA in 401 women with abnormal cytology**

Genotype	Prevalence			Agreement					
	CLART, N (%)	LA, N (%)	Relative prevalence CLART vs. LA (95% CI)	CLART+/LA+	CLART+/LA-	CLART-/LA+	CLART-/LA-	Total agreement (95% CI)	Positive agreement (95% CI)
**High-risk**									
16	121 (30%)	126 (31%)	0.96 (0.78-1.18)	120 (30%)	1 (0%)	6 (1%)	274 (68%)	98% (96-99)	94% (89-98)
18	45 (11%)	53 (13%)	0.85 (0.59-1.23)	45 (11%)	0 (0%)	8 (2%)	348 (87%)	98% (96-99)	85% (72-93)
31	64 (16%)	61 (15%)	1.05 (0.76-1.45)	61 (15%)	3 (1%)	0 (0%)	337 (84%)	99% (98-100)	95% (87-99)
33	42 (10%)	40 (10%)	1.05 (0.70-1.58)	39 (10%)	3 (1%)	1 (0%)	358 (89%)	99% (97-100)	91% (78-97)
35	19 (5%)	16 (4%)	1.19 (0.62-2.28)	16 (4%)	3 (1%)	0 (0%)	382 (95%)	99% (98-100)	84% (60-97)
39	22 (5%)	48 (12%)	0.46 (0.28-0.74)	22 (5%)	0 (0%)	26 (6%)	353 (88%)	94% (91-96)	46% (31-61)
45	16 (4%)	35 (9%)	0.46 (0.26-0.81)	16 (4%)	0 (0%)	19 (5%)	366 (91%)	95% (93-97)	46% (29-63)
51	47 (12%)	50 (12%)	0.94 (0.65-1.37)	46 (11%)	1 (0%)	4 (1%)	350 (87%)	99% (97-100)	90% (79-97)
52	49 (12%)	40 (10%)	1.23 (0.83-1.82)	33 (8%)	16 (4%)	7 (2%)	345 (86%)	94% (92-96)	59% (45-72)
56	30 (7%)	39 (10%)	0.77 (0.49-1.21)	27 (7%)	3 (1%)	12 (3%)	359 (90%)	96% (94-98)	64% (48-78)
58	59 (15%)	40 (10%)	1.48 (1.01-2.15)	38 (9%)	21 (5%)	2 (0%)	340 (85%)	94% (92-96)	62% (49-74)
59	27 (7%)	29 (7%)	0.93 (0.56-1.54)	20 (5%)	7 (2%)	9 (2%)	365 (91%)	96% (94-98)	56% (38-72)
68	25 (6%)	16 (4%)	1.56 (0.85-2.88)	6 (1%)	19 (5%)	10 (2%)	366 (91%)	93% (90-95)	17% (7-34)
**Low-risk**									
6	15 (4%)	13 (3%)	1.15 (0.56-2.39)	12 (3%)	3 (1%)	1 (0%)	385 (96%)	99% (97-100)	75% (48-93)
11	4 (1%)	4 (1%)	1.00 (0.25-3.97)	4 (1%)	0 (0%)	0 (0%)	397 (99%)	100% (99-100)	100% (40-100)
26	3 (1%)	3 (1%)	1.00 (0.20-4.93)	3 (1%)	0 (0%)	0 (0%)	398 (99%)	100% (99-100)	100% (29-100)
40	5 (1%)	6 (1%)	0.83 (0.26-2.71)	5 (1%)	0 (0%)	1 (0%)	395 (99%)	100% (99-100)	83% (36-100)
42	20 (5%)	28 (7%)	0.71 (0.41-1.25)	20 (5%)	0 (0%)	8 (2%)	373 (93%)	98% (96-99)	71% (51-87)
53	50 (12%)	52 (13%)	0.96 (0.67-1.38)	48 (12%)	2 (0%)	4 (1%)	347 (87%)	99% (97-99)	89% (77-96)
54	15 (4%)	36 (9%)	0.42 (0.23-0.75)	14 (3%)	1 (0%)	22 (5%)	364 (91%)	94% (92-96)	38% (22-55)
61	34 (8%)	44 (11%)	0.77 (0.50-1.18)	34 (8%)	0 (0%)	10 (2%)	357 (89%)	98% (95-99)	77% (62-89)
62	12 (3%)	30 (7%)	0.40 (0.21-0.77)	12 (3%)	0 (0%)	18 (4%)	371 (93%)	96% (93-97)	40% (23-59)
66	44 (11%)	40 (10%)	1.10 (0.73-1.65)	37 (9%)	7 (2%)	3 (1%)	354 (88%)	98% (95-99)	79% (64-89)
70	28 (7%)	24 (6%)	1.17 (0.69-1.98)	24 (6%)	4 (1%)	0 (0%)	373 (93%)	99% (97-100)	86% (67-96)
71	1 (0%	2 (0%)	0.50 (0.05-5.49)	0 (0%)	1 (0%)	2 (0%)	398 (99%)	99% (98-100)	0% (0-71)
72	1 (0%)	1 (0%)	1.00 (0.06-15.93)	1 (0%)	0 (0%)	0 (0%)	400 (100%)	100% (99-100)	100% (3-100)
73	11 (3%)	31 (8%)	0.35 (0.18-0.70)	11 (3%)	0 (0%)	20 (5%)	370 (92%)	95% (92-97)	35% (19-55)
81	12 (3%)	14 (3%)	0.86 (0.40-1.83)	12 (3%)	0 (0%)	2 (0%)	387 (97%)	100% (98-100)	86% (57-98)
82	47 (12%)	21 (5%)	2.24 (1.36-3.67)	20 (5%)	27 (7%)	1 (0%)	353 (88%)	93% (90-95)	42% (28-57)
83	15 (4%)	14 (3%)	1.07 (0.52-2.19)	12 (3%)	3 (1%)	2 (0%)	384 (96%)	99% (97-100)	71% (44-90)
84	15 (4%)	26 (6%)	0.58 (0.31-1.07)	15 (4%)	0 (0%)	11 (3%)	375 (94%)	97% (95-99)	58% (37-77)

**Table 3:** **Detection of individual high-risk HPV genotypes by CLART and LA in 125 women with ≥ CIN2**

High-risk genotype	Prevalence			Agreement					
	CLART, N (%)	LA, N (%)	Relative prevalence CLART vs. LA (95% CI)	CLART+/LA+	CLART+/LA-	CLART-/LA+	CLART-/LA-	Total agreement (95% CI)	Positive agreement (95% CI)
16	55 (44%)	57 (46%)	0.96 (0.73-1.27)	55 (44%)	0 (0%)	2 (2%)	68 (54%)	98% (94-100)	96% (88-100)
18	18 (14%)	18 (14%)	1.00 (0.55-1.83)	18 (14%)	0 (0%)	0 (0%)	107 (86%)	100% (97-100)	100% (81-100)
31	30 (24%)	30 (24%)	1.00 (0.64-1.55)	30 (24%)	0 (0%)	0 (0%)	95 (76%)	100% (97-100)	100% (88-100)
33	21 (17%)	22 (18%)	0.95 (0.55-1.64)	21 (17%)	0 (0%)	1 (1%)	103 (82%)	99% (96-100)	95% (77-100)
35	7 (6%)	6 (5%)	1.17 (0.40-3.37)	6 (5%)	1 (1%)	0 (0%)	118 (94%)	99% (96-100)	86% (42-100)
39	4 (3%)	12 (10%)	0.33 (0.11-1.01)	4 (3%)	0 (0%)	8 (6%)	113 (90%)	94% (88-97)	33% (10-65)
45	6 (5%)	17 (14%)	0.35 (0.14-0.87)	6 (5%)	0 (0%)	11 (9%)	108 (86%)	91% (85-96)	35% (14-62)
51	15 (12%)	17 (14%)	0.88 (0.46-1.69)	15 (12%)	0 (0%)	2 (2%)	108 (86%)	98% (94-100)	88% (64-99)
52	17 (14%)	15 (12%)	1.13 (0.59-2.17)	11 (9%)	6 (5%)	4 (3%)	104 (83%)	92% (86-96)	52% (30-74)
56	6 (5%)	9 (7%)	0.67 (0.24-1.82)	5 (4%)	1 (1%)	4 (3%)	115 (92%)	96% (91-99)	50% (19-81)
58	19 (15%)	10 (8%)	1.90 (0.92-3.92)	10 (8%)	9 (7%)	0 (0%)	106 (85%)	93% (87-97)	53% (29-76)
59	9 (7%)	10 (8%)	0.90 (0.38-2.14)	8 (6%)	1 (1%)	2 (2%)	114 (91%)	98% (93-100)	73% (39-94)
68	7 (6%)	3 (2%)	2.33 (0.62-8.82)	0 (0%)	7 (6%)	3 (2%)	115 (92%)	92% (86-96)	0% (0-31)

**Table 4:** **Agreement between CLART and LA with respect to single and multiple infections in 401 women with abnormal cytology**

LA	CLART			Total
	Single infection	Multiple infection	No HPV genotype^a^	
**Single infection**	89	19	13	121 (30%)
**Multiple infection**	40	216	3	259 (65%)
**No HPV genotype** ^**a**^	1	0	20	21 (5%)
**Total**	130 (32%)	235 (59%)	36 (9%)	401 (100%)

^a^Or genotypes not detectable by both CLART and LA (CLART: 43, 44, 85, 89; LA: 55, 64, 67, 69, IS39, CP6108).

**Table 5:** **Agreement between CLART and LA in 401 women with abnormal cytology**

LA	CLART			Total
	High-risk genotypes	Low-risk genotypes	No genotypes^a^	
**High-risk genotypes**	306	12	8	326 (81%)
**Low-risk genotypes**	5	41	8	54 (13%)
**No genotypes** ^**a**^	0	1	20	21 (5%)
**Total**	311 (78%)	54 (13%)	36 (9%)	401 (100%)

^a^Or genotypes not detectable by both CLART and LA (CLART: 43, 44, 85, 89; LA: 55, 64, 67, 69, IS39, CP6108).

The agreement with HC2 in detecting high-risk HPV infections was lower for both genotyping assays (Table 6): for CLART, κ = 0.45, overall agreement = 84% (95% CI: 80–87), and for LA, κ = 0.51, overall agreement 87% (95% CI: 84–90). Of the 355 HC2-positive samples, CLART detected only low-risk genotypes on 43 (12%), and no genotypes on 11 (3%). For LA, this was 36 (10%) and 4 (1%), respectively. Not surprisingly, the agreement with HC2 was better in women with ≥ CIN2.

**Table 6:** **CLART and LA: agreement with HC2**

Genotyping assay	Assay+/HC2+	Assay+/HC2-	Assay-/HC2+	Assay-/HC2-
**401 women with abnormal cytology**				
CLART	301 (75%)	10 (2%)	54 (13%)	36 (9%)
LA	315 (79%)	11 (3%)	40 (10%)	35 (9%)
**125 women with ≥ CIN2**				
CLART	116 (93%)	0 (0%)	7 (6%)	2 (2%)
LA	120 (96%)	0 (0%)	3 (2%)	2 (2%)

### Detection of cervical lesions

CLART detected 116 of 125 ≥ CIN2 (sensitivity: 93%, 95% CI: 87–97), and 84 of 90 ≥ CIN3 (sensitivity: 93%, 95% CI: 86–98; Table 7). LA detected 120 ≥ CIN2 (sensitivity: 96%, 95% CI: 91–99) and 87 ≥ CIN3 (sensitivity: 97%, 95% CI: 91–99). HC2 detected 123 ≥ CIN2 and 89 ≥ CIN3, sensitivity 98% (95% CI: 94–100), and 99% (95% CI: 94–100), respectively. These differences, assessed through relative sensitivity (Table 7), were not statistically significant. Three women with cervical cancer tested positive for high-risk HPV on all three assays. The fourth woman tested negative on CLART, and positive on LA (genotype 39) and HC2. Given that all women had cytological abnormalities, the specificity of all three assays was low, but significantly higher (assessed through relative specificity) for CLART (30%, 95% CI: 25–36, for ≥ CIN2) and LA (26%, 95% CI: 21–32) than for HC2 (17%, 95% CI: 12–22).

**Table 7:** **CLART, LA, and HC2: sensitivity and specificity for ≥ CIN2 and ≥ CIN3**

	CLART	LA	HC2
**Endpoint: ≥CIN2**			
Sensitivity (95% CI)	0.93 (0.87-0.97)	0.96 (0.91-0.99)	0.98 (0.94-1.00)
Relative sensitivity vs. LA	0.97 (0.91-1.03)	1.0 (ref)	1.03 (0.98-1.07)
Relative sensitivity vs. HC2	0.94 (0.89-1.00)	0.98 (0.94-1.02)	1.0 (ref)
Specificity	0.30 (0.25-0.36)	0.26 (0.21-0.32)	0.17 (0.12-0.22)
Relative Specificity vs. LA	1.16 (0.88-1.54)	1.0 (ref)	0.64 (0.46-0.90)
Relative Specificity vs. HC2	1.81 (1.30-2.52)	1.56 (1.11-2.19)	1.0 (ref)
**Endpoint ≥ CIN3**			
Sensitivity (95% CI)	0.93 (0.86-0.98)	0.97 (0.91-0.99)	0.99 (0.94-1.00)
Relative sensitivity vs. LA	0.97 (0.90-1.03)	1.0 (ref)	1.02 (0.98-1.07)
Relative sensitivity vs. HC2	0.94 (0.89-1.00)	0.98 (0.94-1.02)	1.0 (ref)
Specificity	0.28 (0.23-0.33)	0.23 (0.19-0.29)	0.15 (0.11-0.20)
Relative Specificity vs. LA	1.17 (0.89-1.55)	1.0 (ref)	0.64 (0.45-0.90)
Relative Specificity vs. HC2	1.84 (1.32-2.56)	1.57 (1.11-2.21)	1.0 (ref)

## Discussion

In Danish women with abnormal cytology, CLART and LA were highly comparable for detection of HPV genotypes 16, 18, 31, 33, 35, and 51. Furthermore, “substantial” agreement was observed for HPV 39, 45, 52, 56, 58, and 59, which translated into ~50-60% of cases mutually detected by the two assays. Finally, the agreement was poor for HPV 68, present in <1% of cervical cancers [5]. There were no statistically significant differences in detecting high-risk HPV infections overall, and the two assays detected similar numbers of high-grade CIN. For low-risk genotypes, the differences were somewhat more pronounced, but still generally acceptable, although for genotypes HPV 54, 62, 71, 73, and 82 the agreement was poor. The agreement in detecting HPV infections with HC2, a thoroughly validated clinical screening assay [18], was moderate for both CLART and LA, but with no statistically significant differences in detecting high-grade CIN.

CLART was previously compared to LA using ThinPrep samples. Using 538 samples from women in opportunistic examination, Chranioti and colleagues found high levels of agreement in detecting the 13 high-risk HPV genotypes [19]. HPV 68 was though detected in only two samples, in which it was detected by both assays. Analytical performance of CLART and LA was reported as part of the WHO HPV LabNet Proficiency Studies [4,11,20]. In the most recent published evaluation [4], both assays had high analytical sensitivity for HPV 16 and 18, even at low plasmid concentration levels (5–50 international units/genomic equivalent). CLART more often correctly detected HPV 6, 11, 31, 33, 35, 51, 52, 58, 59, and 66 than LA, although the number of compared datasets was small. The opposite was observed for HPV 45, 56 and 68b, which were compared at high concentration levels (500 international units/genomic equivalent). Moreover, WHO LabNet panel data from 2011 showed similar performance in genotype detection between CLART and PapilloCheck [4]. Data from the most recent 2013 WHO global proficiency panel are awaited.

Pista and colleagues compared CLART to HC2 in women attending primary and gynecologic outpatient clinics, and found the same sensitivity for ≥ CIN2, 96%, with similar specificities (74% vs. 71%) [14]. In a study of women referred for colposcopy reported by Szarewski and colleagues, the sensitivity of LA for ≥ CIN2 was 98%, and that of HC2 100%. CLART did not perform optimally owing to “technical problems during the evaluation”, with sensitivity for ≥ CIN2 of only 81%. However, it should be noted that the study used an earlier version of the CLART assay. Furthermore, the accuracy of HPV test results using genotyping assays may improve with a laboratory’s experience with a particular assay, and differences between laboratories can be substantial [4].

Our study is the first comparison of genotype detection and clinical performance of CLART and LA using SurePath samples. It is also one of the first reports on LA with SurePath in general. Previously, Chernesky and colleagues studied 133 routine samples and found a 94% overall agreement in detecting high-risk HPV infections between LA and HC2, with κ = 0.86 [21]. This was substantially higher than in our study; however, the samples in the study by Chernesky and colleagues were tested in two laboratories, and the distribution of cytological abnormalities, an important determinant of agreement between HPV assays [15] was not reported.

One of the strengths of this study was the use of fresh, routine SurePath cytology samples from a large Danish cervical screening laboratory with well-established cytology performance. The genotyping assays were compared on equal terms: all testing was undertaken in the same laboratory by the same staff; samples were processed manually, and the analysis was limited to the 31 HPV genotypes that are detectable by both assays. Each LA hybridization strip was read by two experienced staff members, and discrepancies were resolved by consensus. Histological diagnoses were available for 86% of the women, with only 4% lost to follow-up.

Interpretation of the detection of genotypes with weak signals on both CLART and LA might be considered a weakness of our study in the sense that other laboratories may have opted for different approaches. Our current clinical standard operating procedure calls for weak signals (below cut off, but visible) to be considered positive if the weak signal is detected along with other genotypes detected above the cut off. This approach was though not playing a major role in our data; after including genotypes with weak signals, the test results changed from low-risk to high-risk positive in 7 samples for CLART, and 5 for LA, with 1 and 0 ≥ CIN2, respectively. This low number of ≥ CIN2 was consistent with previous observations of low numbers of CIN lesions found close to manufacturer-determined cut-offs for other HPV assays [22]. The LA and CLART package inserts do not provide information on how to interpret genotype findings with weak signals, leaving it up to the individual laboratory to establish their own interpretation algorithms. Our approach is justified by the fact that individual genotypes appear more difficult to detect in co-infections, as compared to single infections [4], owing to the dynamics of multiplex PCR reactions. In particular, HPV genotypes with high viral loads may through primer or reagent competition lessen the PCR amplification of other truly present genotypes in a sample. This issue and its consequences for the clinical management have been little discussed in the literature.

For use of any HPV assay in cervical screening, quality control and quality assurance aspects should also be considered. In this respect, sample identification is not provided for the individual LA strips, whereas CLART’s software stores sample-specific identification information printed on the individual array by the manufacturer, which is reported alongside the testing results from the automated reader. Hence, CLART has a state-of-the-art chain of custody and is not prone to inter-observer variability given the automated read-out.

## Conclusions

In our referral population, CLART was comparable to LA in terms of analytical and clinical performance, and CLART’s clinical sensitivity was comparable to that of HC2, whereas its specificity was higher. In the absence of an internationally recognized genotyping gold standard, CLART HPV2 appears to be a good candidate for genotyping HPV infections in clinical settings where high throughput and chain of custody is required.

## Abbreviations

AGC: Atypical glandular cells
AIS: Adenocarcinoma in situ
ASC-H: Atypical squamous cells – cannot exclude HSIL
ASCUS: Atypical squamous cells of undetermined significance
CI: Confidence interval
CIN: Cervical intraepithelial neoplasia
HC2: Hybrid capture 2
HPV: Human papillomavirus
HSIL: High-grade squamous intraepithelial lesions
LA: Linear array
LSIL: Low-grade squamous intraepithelial lesions
PCR: Polymerase chain reaction
RP: Relative proportion

## References

[1] Bouvard V, Baan R, Straif K, Grosse Y, Secretan B, El Ghissassi F (2009). A review of human carcinogens–Part B: biological agents. Lancet Oncol.

[2] Garland SM, Steben M, Sings HL, James M, Lu S, Railkar R (2009). Natural history of genital warts: analysis of the placebo arm of 2 randomized phase III trials of a quadrivalent human papillomavirus (types 6, 11, 16, and 18) vaccine. J Infect Dis.

[3] Walboomers JM, Jacobs MV, Manos MM, Bosch FX, Kummer JA, Shah KV (1999). Human papillomavirus is a necessary cause of invasive cervical cancer worldwide. J Pathol.

[4] Eklund C, Forslund O, Wallin KL, Dillner J (2014). Global improvement in genotyping of human papillomavirus DNA: the 2011 HPV LabNet International Proficiency Study. J Clin Microbiol.

[5] Li N, Franceschi S, Howell-Jones R, Snijders PJ, Clifford GM (2011). Human papillomavirus type distribution in 30,848 invasive cervical cancers worldwide: Variation by geographical region, histological type and year of publication. Int J Cancer.

[6] Didelot MN, Boulle N, Damay A, Costes V, Segondy M (2011). Comparison of the PapilloCheck(R) assay with the digene HC2 HPV DNA assay for the detection of 13 high-risk human papillomaviruses in cervical and anal scrapes. J Med Virol.

[7] Hesselink AT, Heideman DA, Berkhof J, Topal F, Pol RP, Meijer CJ (2010). Comparison of the clinical performance of PapilloCheck human papillomavirus detection with that of the GP5+/6 + −PCR-enzyme immunoassay in population-based cervical screening. J Clin Microbiol.

[8] Iftner T, Eberle S, Iftner A, Holz B, Banik N, Quint W (2010). Prevalence of low-risk and high-risk types of human papillomavirus and other risk factors for HPV infection in Germany within different age groups in women up to 30 years of age: an epidemiological observational study. J Med Virol.

[9] Galan-Sanchez F, Rodriguez-Iglesias MA (2009). Comparison of human papillomavirus genotyping using commercial assays based on PCR and reverse hybridization methods. APMIS.

[10] Perez C, Klaustermeier JE, Alemany L, Tous S, de Sanjosé S, Velasco J (2012). Comparison of 2 different PCR-based technologies for the detection of human papilloma virus from paraffin-embedded tissue: genomica clinical arrays versus SPF(10)-LiPA(25). Diagn Mol Pathol.

[11] Eklund C, Forslund O, Wallin KL, Zhou T, Dillner J, WHO Human Papillomavirus Laboratory Network (2012). The 2010 global proficiency study of human papillomavirus genotyping in vaccinology. J Clin Microbiol.

[12] Bonde J, Rebolj M, Ejegod DM, Preisler S, Lynge E, Rygaard C (2014). HPV prevalence and genotype distribution in a population-based split-sample study of well-screened women using CLART HPV2 human papillomavirus genotype microarray system. BMC Infect Dis.

[13] Mejlhede N, Pedersen BV, Frisch M, Fomsgaard A (2010). Multiple human papilloma virus types in cervical infections: competition or synergy?. APMIS.

[14] Pista A, Verdasca N, Oliveira A (2011). Clinical performance of the CLART human papillomavirus 2 assay compared with the hybrid capture 2 test. J Med Virol.

[15] Rebolj M, Preisler S, Ejegod DM, Rygaard C, Lynge E, Bonde J (2014). Disagreement between human papillomavirus assays: an unexpected challenge for the choice of an assay in primary cervical screening. PLoS One.

[16] Bjerregaard B, Larsen OB (2011). The Danish Pathology Register. Scand J Public Health.

[17] Landis JR, Koch GG (1977). The measurement of observer agreement for categorical data. Biometrics.

[18] Ronco G, Dillner J, Elfström KM, Tunesi S, Snijders PJ, Arbyn M (2014). Efficacy of HPV-based screening for prevention of invasive cervical cancer: follow-up of four European randomised controlled trials. Lancet.

[19] Chranioti A, Spathis A, Aga E, Meristoudis C, Pappas A, Panayiotides I (2012). Comparison of two commercially available methods for HPV genotyping: CLART HPV2 and Linear Array HPV Genotyping tests. Anal Quant Cytol Histol.

[20] Eklund C, Zhou T, Dillner J (2010). Global proficiency study of human papillomavirus genotyping. J Clin Microbiol.

[21] Chernesky M, Jang D, Portillo E, Smieja M, Chong S, Buracond S (2011). Comparative evaluation of AMPLICOR HPV PCR and Linear Array assays on SurePath liquid-based Pap samples for the detection of high-risk HPV genotypes. J Clin Virol.

[22] Rebolj M, Bonde J, Njor SH, Lynge E (2011). Human papillomavirus testing in primary cervical screening and the cut-off level for hybrid capture 2 tests: systematic review. BMJ.

